# The Effects of Organizational-Level Factors on the National Disability Prevention Programs for Older Adults

**DOI:** 10.1093/geroni/igaa057.267

**Published:** 2020-12-16

**Authors:** Ya-Mei Chen, Wan-Yu Chiu, Hui-Fen Mao, Ling-Hui Chang

**Affiliations:** 1 National Taiwan University, Taipei, Taiwan (Republic of China); 2 National Cheng Kung University, Tainan, Taiwan (Republic of China)

## Abstract

In 2017, Taiwan implemented nationwide disability preventive programs that aimed to maintain older adults’ function and prevent disability. This study aimed to evaluate the impact of organizational-level factors on older adults’ improvement through participating in the 2019 preventive disability programs. There were 202 disability preventive programs, which included a 2-hour sessions per week for 12 weeks, in Taiwan in 2019. Participants’ physical, mental, and social functions were assessed before and after participating the programs and reported to the government. A total of 7194 older adults’ assessments were received. This study administered survey by the end of 2019 in the 202 programs in Taiwan and 8 organizational-level factors were assessed, including effectiveness, efficiency, professional, support from the upper organization, and cooperation with community, adequacy, responsiveness, and sustainability. Multilevel analysis was conducted to assess the impacts of organizational-level factors on disability prevention programs. A total of 170 programs (84%) has responded to our survey. About 48.2% of the programs were implemented by local public health centers. The domains of responsiveness and sustainability are rated highest among these programs. Programs with the following characteristics were associated with better program outcomes among older adults: better ability to provide outreach services was associated with improved depression; higher level in profession domain was associated with improved f mobility; better program responsiveness was associated with improved in nutritional status and oral function, and depression risk. Our results shed lights on how to improve a national disability prevention programs from programs’ characteristics point of view.

